# Metabolic engineering of *Serratia marcescens* MG1 for enhanced production of (*3R*)-acetoin

**DOI:** 10.1186/s40643-016-0128-2

**Published:** 2016-11-28

**Authors:** Xin Lv, Lu Dai, Fangmin Bai, Zhanqing Wang, Liaoyuan Zhang, Yaling Shen

**Affiliations:** 1 State Key Laboratory of Bioreactor Engineering, Shanghai Collaborative Innovation Center for Biomanufacturing Technology, East China University of Science and Technology, Shanghai, 200237 China; 2 Key Laboratory of Biopesticide and Chemical Biology, Ministry of Education, College of Life Sciences, Fujian Agriculture and Forestry University, Fuzhou, 350002 Fujian China

**Keywords:** (*3R*)-AC, *Meso*-2, 3-Butanediol dehydrogenase, Glycerol dehydrogenase, Transcriptional regulator, SlaR, *Serratia marcescens* MG1

## Abstract

**Background:**

Optically pure acetoin (AC) is an important platform chemical which has been widely used to synthesize novel optically active α-hydroxyketone derivatives and liquid crystal composites.

**Results:**

In this study, *sla*C and *gld*A encoding *meso*-2,3-butanediol dehydrogenase (*meso*-2,3-BDH) and glycerol dehydrogenase (GDH), respectively, in *S. marcescens* MG1 were knocked out to block the conversion from AC to 2,3-butanediol (2,3-BD). The resulting strain MG14 was found to produce a large amount of optically pure (*3R*)-AC with a little 2,3-BD, indicating that another enzyme responsible for 2,3-BD formation except *meso*-2,3-BDH and GDH existed in the strain MG1. Furthermore, SlaR protein, a transcriptional activator of AC cluster, was overexpressed using *P*_C_ promoter in the strain MG14, leading to enhancement of the (*3R*)-AC yield by 29.91%. The recombinant strain with overexpression of SlaR, designated as *S. marcescens* MG15, was used to perform medium optimization for improving (*3R*)-AC production.

**Conclusion:**

Under the optimized conditions, 39.91 ± 1.35 g/l (*3R*)-AC was produced by strain MG15 with the productivity of 1.11 g/l h and the conversion rate of 80.13%.

## Background

Acetoin (AC), also named as 3-hydroxy-2-butanone, is a widely used platform chemical which has been applied in many fields such as food additive, cosmetic products, pharmaceuticals, and chemical synthesis (Xiao and Xu 2007; Xiao and Lu 2014). (*3R*)-AC and (*3S*)-AC are two stereoisomeric forms of AC, both of which are important pharmaceutical intermediates (Liu et al. 2011a). However, Chiral AC is usually more valuable, while it is often used to synthesize novel optically active α-hydroxyketone derivatives and liquid crystal composites (Xiao et al. 2010).

A lot of microbials have been reported to be capable of producing a large amount of AC, such as *Klebsiella pneumonia* (Wang et al. 2015), *Bacillus subtilis* (Zhang et al. 2013a), *Enterobacter cloacae* (Zhang et al. 2016), and *Paenibacillus polymyxa* (Zhang et al. 2012). However, none of these strains had the ability to produce optically pure (*3R*)-AC or (*3S*)-AC. The current highest production of 75.2 g/l AC was achieved from *Serratia marcescens* H32 without considering its isomer (Sun et al. 2012). Thus, *S. marcescens* was chosen to be the candidate to produce optically pure AC with high concentration in this study.

During the fermentation process, pyruvate produced from the glycolytic pathway was converted into α-acetolactate by α-acetolactate synthase (α-ALS) (Biswas et al. 2012). The majority of α-acetolactate was subsequently transformed into (*3R*)-AC catalyzed by *α*-acetolactate decarboxylase (α-ALDC), while a small amount of α-acetolactate was transformed into diacetyl (DA), by the non-enzymatic oxidation decarboxylation (Liu et al. 2011b; Yang et al. 2014), and DA could be further converted into (*3S*)-AC which decreased the stereoisomeric purity of (*3R*)-AC. Both (*3R*)-AC and (*3S*)-AC were then transformed into 2,3-BD by the *meso*-2,3-BDH and the GDH encoded by *sla*C and *gld*A (Bai et al. 2015; Zhang et al. 2014), respectively, resulting low AC yield. So the native strain should be genetically modified for the production of optically pure AC.

It was found that the genes encoding α-ALS and α-ALDC usually formed a gene cluster, which was regulated by a transcriptional activator. The *als*SD operon in *Bacillus subtilis* responsible for the expression of α-ALS and α-ALDC was regulated by the transcriptional regulator AlsR which was encoded by *alsR* (Renna et al. 1993; Frädrich et al. 2012), while the AC cluster in *S.marcescens* was found to be regulated by SlaR protein encoded by *slaR* (Rao et al. 2012). Since overexpression of AlsR had been proved to be successful for enhancing AC production by *B.subtilis* (Zhang et al. 2013b), similar protocol was attempted in *S. marcescens* MG1.

In this study, to obtain a (*3R*)-AC producing strain, both the *sla*C and *gld*A were knocked out in *S.marcescens* MG1 using pUTKm1 as the suicide vector. The resulting mutant strain MG14 was found to produce single configuration of optically pure (*3R*)-AC from sucrose. Besides, three endogenous promoters were attempted to express the transcriptional regulator SlaR for the purpose of enhancing the production of (*3R*)-AC. Finally, the optimal culture conditions and the suitable fermentation medium component were developed for the engineered strain using statistical methods.

## Methods

### Enzymes and chemicals

Restriction enzymes were purchased from Thermo Fisher (USA). T4 DNA ligase and Primestar were purchased from TaKaRa Biotech (Dalian, China). Taq DNA polymerase was purchased from CW Bio (Beijing, China). Bacterial DNA kit was purchased from SBS Genetech (Beijing, China), plasmid mini kit, cycle-pure kit, and gel extraction kit were purchased from Omega Biotech (Norcross, America). AC and 2,3-BD were purchased from Sigma-Aldrich (Shanghai, China).

### Constructing of *S.marcescens* ∆*sla*C, *S.marcescens* ∆*gld*A, and *S.marcescens* ∆*sla*C∆*gld*A

The strains, plasmids, and primers used in this study are listed in Table 1. The suicide plasmid pUTKm1 (Lorenzo et al. 1990) was used to knock out *sla*C and *gld*A in *Serrata marcescens* MG1. In the case of *sla*C, the left and right flanking sequences of *sla*C were amplified from the genomic DNA of *S.marcescens* MG1 and then fused by overlap PCR. The ∆*sla*C fragments were gel purified and then ligated into pUTKm1. The resulting plasmid was designated pUT-*sla*C. For conjugation, *E. coli* S17-1 λpir was used as the donor strain to amplify and transfer the suicide plasmid pUT-*sla*C. The conjugation cells were recovered with fresh LB and plated on selection medium plates. One of the kanamycin-resistant transconjugants (single-crossover) was picked on LB plate containing 50 μg/ml kanamycin. The single-crossover was then inoculated at 30 °C for 12 h on LB plate without antibiotic; Kanamycin-sensitive colonies from a double crossover event were verified by PCR using primers slaC-F1 and slaC-R2 to obtain the *sla*C mutant strain MG12. The *gld*A from MG1 and MG12 was knocked out individually in the same way to construct the *gld*A mutant strain MG13 and the double-knockout strain MG14.

**Table 1:** Strains, plasmids, and primers used in this study

Strains, plasmids and primers	Description	Source
*E.coli* S17-1(*λ pir*)	*recA thi pro hsdR* ^−^ *M* ^+^ *RP4::2*-*Tc::Mu::Km Tn7 lysogenized with* λ pir phage	Laboratory stock
*S. marcescens* MG1	Wild type, Tc^r^Ap^r^	Laboratory stock
MG12	*S. marcescens* MG1 Δ*sla*C	This study
MG13	*S. marcescens* MG1 Δ*gld*A	This study
MG14	*S. marcescens* MG1 Δ*slaC*Δ*gld*A	This study
MG15	*S. marcescens* MG1 Δ*sla*CΔ*gld*A/pACP_C_-*sla*R	This study
MG16	*S. marcescens* MG1 Δ*sla*CΔ*gld*A/pACP_AB_-*sla*R	This study
MG17	*S. marcescens* MG1 Δ*sla*CΔ*gld*A/pACP_R_-*sla*R	This study
pUTKm1	Ap^r^ Km^r^ *ori*R6 K *ori*TRP4	(Lorenzo et al. 1990)
pUT-*sla*C	pUTKm1 containing a 1798 bp deletion of *sla*C	This study
pUT-*gld*A	pUTKm1 containing a 1804 bp deletion of *gld*A	This study
pACYC184	Cm^R^	(Sun et al. 2015)
pACP_C_-*sla*R	pACYC184 containing *sla*R under the promotor of *sla*C	This study
pACP_A_-*sla*R	pACYC184 containing *sla*R under the promotor of *sla*A	This study
pACP_R_-*sla*R	pACYC184 containing *sla*R under the promotor of *sla*R	This study
slaC-F1	GTggtaccCATGCGGCAAGGAGCGCCATC	This study
slaC-F2	GGCCTGTGCGTTAACGCGAGACCTCCTCCATGTGAAC	This study
slaC-R1	GTTCACATGGAGGAGGTCTCGCGTTAACGCACAGGCC	This study
slaC-R2	GTgagtactCAGCCGCATCAGCCGCTAC	This study
gldA-F1	TTCggtaccGGTTGCGTTCAATGATGATG	This study
gldA-F2	CTCCCTACAAGGATCCGGTTTACCCTTGGGGCGCGGTGTGC	This study
gldA-R1	GCACACCGCGCCCCAAGGGTAAACCGGATCCTTGTAGGGAG	This study
gldA-R2	GCTagatctCTGCATGCTGGTCTGCTTGG	This study
*P*_C_-1	GTtctagaTCGCGGCCGCCTGCGGGC	This study
*P*_C_-2	GTaagcttGAGACCTCCTCCATGTG	This study
*P*_A_-1	GCtctagaAAAACGTAATATACGTTT	This study
*P*_A_-1	GCaagcttCTGACTGAGGAGGTGGTC	This study
*P*_R_-1	GCtctagaCTGACTGAGGAGGTGGTCGC	This study
*P*_R_-2	GCaagcttTTTTGCATTATATGCAAA	This study
slaR-1	GCaagcttATGAATGACGCACGCTATG	This study
slaR-2	GCggatccAATAGGGGTCGACCCGCCAA	This study

### Expression of *sla*R with different promoters in engineered strain

The construction of recombinant plasmids is also shown in Table 1. The *sla*R gene encoding the transcriptional regulator SlaR was amplified from the genomic DNA of *S.marcescens* MG1 using primers slaR-1 and slaR-2. The expression plasmid PAC-*sla*R was constructed by inserting gene slaR into pACYC184 (Sun et al. 2015) between the *Hind*III and *Bam*HI sites. The cassettes of promoter *P*_C_ (promoter of *sla*C), *P*_A_ (promoter of *sla*A), and *P*_R_ (promoter of *sla*R) were amplified from the genomic DNA of MG1 using primer pairs *P*_C_-1/*P*_C_-2, *P*_A_-1/*P*_A_-2, and *P*_R_-1/*P*_R_-2. The three purified PCR products were double-digested with *Xba*I/*Hind*III and ligated to the corresponding sites of pAC-slaR, respectively, to generate pAC-*P*_C_-*sla*R, pAC-*P*_AB_-*sla*R, and pAC-*P*_R_-*sla*R. The vectors were then introduced into *S. marcescens* MG14 separately by electroporation. The recombinants were selected on LB plates supplemented with 25 μg/ml chloramphenicol and designated as MG15, MG16, and MG17, respectively.

### Enzyme activity assays

The engineered strains were grown at 30  C for 10 h, and then the fermentation broth was centrifuged at 8000 rpm for 10 min. After washing twice and resuspending with 50 mM phosphate buffer (pH 7.0), the cells were disrupted with an ultrasonic cell breaking apparatus (Xinzhi, Ningbo, China). The resulting supernatant was finally obtained through centrifugation and was used for assaying enzyme activities (Kousoulos et al. 2006).

The AC reductase (AR) activities were determined by measuring NADH oxidation at 340 nm using a UV/visible spectrophotometer (UV-7504, Xin Mao, China). (Wayne 2008; Hao et al. 2014). The reaction was initiated by adding 20 μl crude extract to 1 ml reaction buffer which contained 67 mM phosphate buffer (pH 7.4), 5 mM of AC, and 0.2 mM of NADH. One unit of AR activity was defined as the amount of enzyme that consumed 1 μM of NADH per min at 30  C.

To determine the integrated activities of α-ALS and α-ALDC, the reaction was initiated by addition of 100 μl of crude extract to 3 ml reaction buffer containing 100 mM potassium phosphate buffer (pH 6.5), 20 mM sodium pyruvate, 0.01 mM flavin adenine dinucleotide (FAD), 0.5 mM thiamine pyrophosphate (TPP), and 0.5 mM MgCl_2,_ and then the reaction was terminated by adding 0.5 ml 2 M NaOH. One unit of the integrated activity of α-ALS and α-ALDC was defined as the amount of enzyme required for the formation of 1 μM AC per milligram protein per minute at 30 °C. Total protein concentrations were determined using the Bradford method described by Marion (1976).

### Analytical methods

The cell density was monitored by determining the absorbance at 600 nm using a spectrophotometer (UV-2008 h, Unic). To determine the residual sucrose concentrations of the samples, the sucrose of the samples was hydrolyzed to glucose by 2 M H_2_SO_4_ and determined using a bioanalyzer (SBA-40D, Shandong Academy of Sciences, China) after centrifugation. The intracellular NADH and NAD^+^ concentrations were determined by procedures described in the previous studies (Snoep et al. 1991). Extracellular metabolites were measured using an HPLC (Agilent 1100) system equipped with an SB-Aq C18 (4.6 mm × 250 mm) column. The column temperature was 30 °C, and the mobile phase was 0.01 M KH_2_PO_4_ (pH = 2.12) with the flow rate at 0.6 ml/min. AC and 2,3-BD in the fermentation broth were extracted by ethyl acetate with the addition of isoamyl alcohol as internal standard and then quantified by GC (Agilent GC9860) equipped with a chiral column (FID-detector, Supelco β-DEX™ 120, 30 m length, 0.25 mm inner diameter). The operation conditions used were as follows: *N*_2_ was used as the carrier gas at a flow rate of 1.2 ml/min; the injector temperature and the detector temperature were 215 and 245 °C, respectively; the oven temperature was maintained at 50 °C for 1.5 min, and then raised to 180 °C at a rate of 8 °C/min. A calibration curve was used to determine the concentration of the products.

### Medium and growth conditions

Luria–Bertani (LB) medium of 50 ml in 250 ml flask was used for culturing *E. coli*, *S. marcescens* MG1, and its derivatives on a rotary shaker at 200 rpm. *E. coli* was grown at 37 °C and *S. marcescens* MG1 was grown at 30 °C. If necessary, the LB medium was supplemented with 100 μg/ml ampicillin, 50 μg/ml kanamycin, or 25 μg/ml chloramphenicol.

For fermentation experiments, seeds cultivation was conducted in 250 ml shake flasks containing 30 ml fresh seed culture for 12 h and then inoculated (5%, v/v) into a 250 ml shake flask with 50 ml of fermentation medium at 30 °C on a rotary shaker at 200 rpm. The seed medium was composed of the following (g/l): glucose 10, peptone 2, yeast extract 1, (NH_4_)_2_SO_4_ 6, KH_2_PO_4_ 10, NaCl 0.5, and MgSO_4_ 0.5 at pH 7.2. The initial fermentation medium was composed of the following (g/l): sucrose 90, yeast extract 25, sodium citrate 14, sodium acetate 4, NH_4_H_2_PO_4_ 3, MgSO_4_ 0.5, and MnSO_4_ 0.05 (Rao et al. 2012).

### Optimization of culture conditions and medium composition

For culture conditions, the effects of temperature (28, 30, 33, 35, 37 °C), pH (6.0, 6.5, 7, 7.5, and 8.0), liquid volume (10, 20, 30, 40, and 50 ml), and inoculation size (1, 3, 5, 7, and 9%, v/v) on (*3R*)-AC production on engineered *S. marcescens* were investigated. The whole fermentation process was conducted for 36 h. The optimum culture conditions were chosen for experiments of medium optimization.

For medium optimization, sucrose, yeast extract, sodium citrate, sodium acetate, NH_4_H_2_PO_4_, MgSO_4_, and MnSO_4_ were chosen for further optimization based on our preliminary experiments. Plackett–Burman (PB) design was used to select factors that significantly influenced (*3R*)-AC production. Table 2 lists the levels of the seven variables and Table 3 shows the design details and responsible results. Variables with significant effects on (*3R*)-AC production were screened from PB design for further optimization. Response surface methodology (RSM) based on central composite design (CCD) was conducted to determine the optimal levels of the variables with significant effects for improving (*3R*)-AC production. The design details are given in Table 4. The experimental designs and analysis were conducted with Minitab 15.0 (Minitab Inc., State College, PA, USA). All experiments were repeated three times.

**Table 2:** The Plackett–Burman design for screening variables in (*3R*)-AC production

Factors (g/l)	Variables	Low level (−1)	High level (+1)	Effect	Coef.	*t* value	*p* value
Sucrose	*X*_1_	60	90	3.310	0.322	10.29	0.001
Yeast extract	*X*_2_	15	25	0.287	0.322	0.89	0.423
Sodium citrate	*X*_3_	5	15	0.602	0.322	1.87	0.135
Sodium acetate	*X*_4_	1	3	−1.012	0.322	−3.14	0.035
NH_4_H_2_PO_4_	*X*_5_	0.2	0.5	0.628	0.322	1.95	0.123
MgSO_4_	*X*_6_	0.2	0.5	−0.057	0.322	−0.18	0.869
MnSO_4_	*X*_7_	0.02	0.05	−0.842	0.322	−2.62	0.059

*R* ^2^ = 97.03%, *R* ^2^(adj) = 91.83%

*Coef* coefficient

**Table 3:** The Plackett–Burman design variables (in coded levels) with (*3R*)-AC as response

Run	Variable levels							(*3R*)-AC (g/l)
	*X*_1_	*X*_2_	*X*_3_	*X*_4_	*X*_5_	*X*_6_	*X*_7_	
1	−1	−1	1	1	1	−1	1	20.68
2	−1	−1	−1	1	1	1	−1	20.67
3	−1	1	−1	−1	−1	1	1	19.78
4	1	−1	1	1	−1	1	−1	26.76
5	1	−1	−1	−1	1	1	1	27.78
6	1	−1	1	−1	−1	−1	1	27.69
7	1	1	−1	1	1	−1	1	26.66
8	1	1	−1	1	−1	−1	−1	26.80
9	−1	1	1	−1	1	−1	−1	23.81
10	−1	−1	−1	−1	−1	−1	−1	22.84
11	1	1	1	−1	1	1	−1	32.31
12	−1	1	1	1	−1	1	1	20.50

**Table 4:** The design and results based on CCD

Run	Sucrose		Sodium acetate		(*3R*)-AC (g/l)
	Code *X*_1_	*X*_1_ (g/l)	Code *X*_2_	*X*_2_ (g/l)	
1	0	80.00	0	2.00	33.23
2	0	80.00	0	2.00	33.79
3	1	100.00	1	3.00	34.43
4	1.41421	108.284	0	2.00	38.89
5	−1	60.00	1	3.00	24.96
6	0	80.00	−1.41421	0.58579	34.2
7	−1.41421	51.716	0	2.00	25.06
8	0	80.00	1.41421	3.41421	31.06
9	0	80.00	0	2.00	34.83
10	1	100.00	−1	1.00	39.48
11	−1	60.00	−1	1.00	29.3
12	0	80.00	0	2.00	34.51
13	0	80.00	0	2.00	33.84

## Results and discussion

### Growth and metabolic profiles of *S. marcescens* MG1 and the mutant trains

*Serratia marcescens* was regarded to be a good 2,3-BD-producing strain. As shown in the study of Rao et al. (2012), *S. marcescens* MG1 produced 42.5 g/l 2,3-BD under 24 h of flask fermentation using sucrose as the substrate, while only 4.6 g/l of AC was accumulated during the fermentation process. As the precursor of 2,3-BD, AC was readily converted into 2,3-BD, resulting in AC production with low yield. Two enzymes, *meso*-2,3-BDH and GDH, respectively, encoded by *sla*C and *gld*A were found to be responsible for the reduction of AC to 2,3-BD. Three mutant strains were constructed by knocking out the *sla*C and *gld*A individually or in combination. Fermentation experiments were subsequently conducted to investigate the metabolic profiles of the strains.

**Fig. 1:**
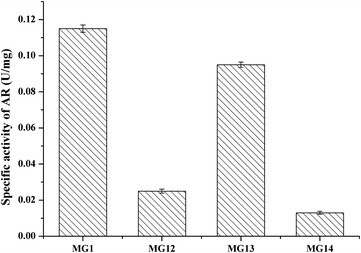
The enzyme activity of AR of *MG1*, *MG12*, *MG13*, and *MG14*

As shown in Fig. 1 and Table 6, deletion of *sla*C or/and *gld*A reduced the AR activity and improved the AC production. (*3R*)-AC concentrations of 19.79 and 6.52 g/l were obtained from strains MG 12 and MG 13, respectively, while only 2.92 g/l (*3R*)-AC was produced from *S. marcescens* MG1 (Table 6). This result demonstrated that the inactivation of *sla*C or *gld*A had positive effects on (*3R*)-AC production. However, lower cell density, longer fermentation time, and lower comprehensive titer of AC and 2,3-BD were observed after *sla*C inactivation. The *sla*C and *gld*A double-knockout strain MG 14 accumulated the highest concentration of (*3R*)-AC without (*3S*)-AC. This indicated that *sla*C and *gld*A were the only two genes responsible for the production of (*3S*)-AC in *S. marcescens* MG1. Therefore, *S. marcescens* MG14 was chosen for further investigation for (*3R*)-AC production.

In addition to AC production, the intracellular NADH and NAD^+^ concentration was also observed. Table 5 shows that disruption of *sla*C and/or *gld*A could increase the NADH/NAD^+^ ratio, especially when *sla*C was knocked out. This was probably because the 2,3-BD pathway which participated in the regulation of NADH/NAD^+^ ratio (Celinska and Grajek 2009) was disturbed, while the synthesis of the by-products such as lactic acid, ethanol, and succinate was NADH-dependent. As shown in Table 6, the concentrations of lactic acid, ethanol, and succinate in MG12 and MG 14 were higher than that in MG1 and MG13. It could be inferred that the higher NADH/NAD^+^ ratio might result in higher by-products concentrations. This could also explain the lower comprehensive titer of AC and 2,3-BD in MG12 and MG14. Besides, a small amount of 2,3-BD was detected in the fermentation broth of MG14, which indicated that another 2,3-BD pathway might exist in *S. marcescens* MG1.

**Table 5:** Intracellular NADH, NAD^+^ and ratios of NADH/NAD^+^ in MG1 and its derivatives

Strains	MG1		MG12		MG13		MG14	
Fermentation time (h)	18	36	18	36	18	36	18	36
Intracellular NADH (μmol/l OD_600_)	0.32 ± 0.01	0.25 ± 0.01	0.41 ± 0.02	0.37 ± 0.02	0.36 ± 0.02	0.33 ± 0.01	0.45 ± 0.02	0.41 ± 0.02
Intracellular NAD^+^ (μmol/l OD_600_)	0.35 ± 0.01	0.28 ± 0.01	0.31 ± 0.01	0.25 ± 0.01	0.33 ± 0.01	0.28 ± 0.01	0.28 ± 0.01	0.25 ± 0.01
Ratio of NADH/NAD^+^	0.91 ± 0.01	0.89 ± 0.01	1.32 ± 0.02	1.48 ± 0.02	1.09 ± 0.02	1.18 ± 0.01	1.61 ± 0.02	1.64 ± 0.02

**Table 6:** Comparison of the fermentation of *S. marcescens* MG1 and its derivatives

Strains	MG1	MG12	MG13	MG14	MG15
Fermentation time (h)	24	36	24	36	36
Consumed sucrose (g/l)	89.2 ± 0.07	87.9 ± 0.14	88.7 ± 0.15	86.3 ± 0.31	96.8 ± 0.65
OD_600_	17.76 ± 0.31	11.63 ± 0.56	17.73 ± 0.22	10.97 ± 0.42	7.28 ± 0.23
(2R,3R)-2,3-BD (g/l)	0.92 ± 0.31	2.96 ± 0.31	0.61 ± 0.31	1.55 ± 0.31	1.85 ± 0.13
(2S,3S)-2,3-BD (g/l)	0.67 ± 0.11	ND^a^	0.71 ± 0.09	ND	ND
*meso*-2,3-BD (g/l)	34.01 ± 1.21	1.28 ± 0.31	31.62 ± 0.98	1.25 ± 0.06	1.52 ± 0.05
(*3R*)-AC (g/l)	2.92 ± 0.08	19.79 ± 0.21	6.52 ± 0.08	21.04 ± 0.31	39.91 ± 1.35
(*3S*)-AC (g/l)	0.79 ± 0.05	0.21 ± 0.03	0.63 ± 0.06	ND	ND
Lactic acid	3.06 ± 0.03	8.05 ± 0.06	3.42 ± 0.03	8.15 ± 0.07	6.83 ± 0.05
Ethanol	0.7 ± 0.01	1.6 ± 0.01	0.6 ± 0.01	1.8 ± 0.01	1.5 ± 0.02
Succinic acid	1.9 ± 0.02	3.8 ± 0.03	2.1 ± 0.02	4.1 ± 0.04	2.6 ± 0.04

^a^Not detected

### Enhanced AC production by overexpression SlaR

SlaR, as the transcriptional regulator of the AC cluster in *S. marcescens* MG1, was reported to be essential for the expression of *sla*A and *sla*B, which direct the synthesis of AC in *S. marcescens* MG1 (Rao et al. 2012). Since moderate expression of the transcription regulator ALsR under P_bdhA_ (promoter of *bdh*A) has been proved to efficiently increase acetoin production in *Bacillus subtilis*, similar procedure was attempted in *Serratia marcescens* MG1. In our previous work, *P*_C_ was proved to be a moderate promoter in MG1 (data not shown). *P*_C_ in MG1 might function similar to P_bdhA_ in *Bacillus subtilis*. *P*_A_ and *P*_R_ were the only two related promoters in the AC operon. In order to find the most suitable promoter, experiments were carried out with these promoters.

**Fig. 2:**
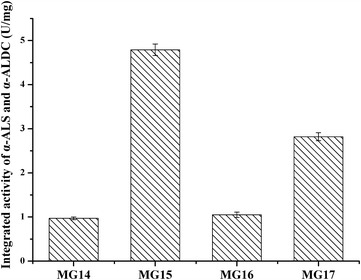
The integrated activity of α-ALS and α-ALDC of *MG14*, *MG 15*, *MG16*, and *MG17*

In this study, three plasmids were constructed for *sla*R expression under the promoter of *P*_C_, *P*_A_, and *P*_R_, respectively. The resulting plasmids pACP_C_-*sla*R, pACP_A_-*sla*R, and pACP_R_-*sla*R were transformed into MG14 by electroporation. The resulting strains were named MG15, MG16, and MG17. The integrated activity of α-ALS and α-ALDC in strains MG14, MG15, MG16, and MG17 was determined at approximately 30 h in flask fermentation (Fig. 2). The results showed that the comprehensive activity of α-ALS and α-ALDC was 4.79, 1.05, and 2.82 U/mg in strains MG15, MG16, and MG17, respectively, while the activity was 0.97 U/mg in strain MG14. These results indicated that the expression of SlaR resulted in more efficient expression of *sla*AB under the control of *P*_C_ than *P*_A_ or *P*_R_, 394% improvement of the integrated activity of α-ALS and α-ALDC was detected in MG15.

**Fig. 3:**
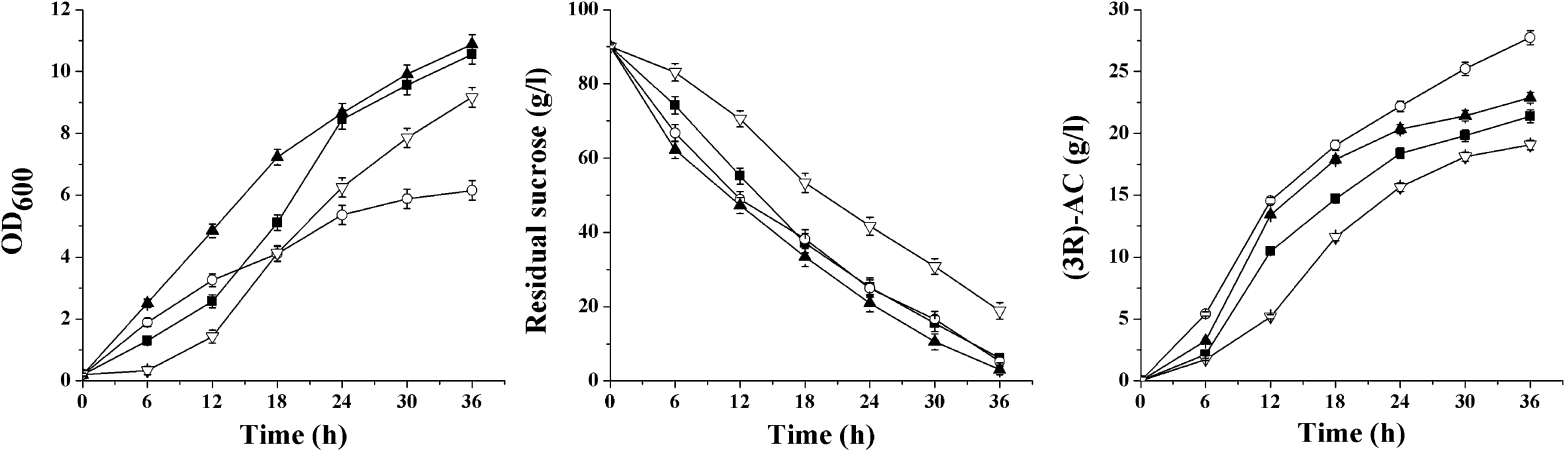
Curves of cell growth, residual sucrose, and (*3R*)-AC production in shake flask fermentation by strain MG14 (*Black square*), MG15 (*White circle*), MG16 (*Black up-pointing triangle*), and MG17 (*White down-pointing triangle*)

As shown in Fig. 3, cell growth, sucrose consumption rate, and (*3R*)-AC production were determined in the engineered strains. Strain MG15 had the lowest cell density (OD_600_ = 6.24), but the highest (*3R*)-AC production of 27.74 g/l, which was 29.91% higher than that of MG14. No obvious difference was detected in cell density and sucrose consumption of MG16 compared to MG14, while the (*3R*)-AC production was improved by 7.07%. Lower cell growth rate and sucrose consumption rate was observed from MG17 than MG14, and the (*3R*)-AC concentration of 19.08 g/l produced by MG17 was the lowest. In summary, the highest (*3R*)-AC production was achieved in MG15, so *P*_C_ was chosen as the most suitable promoter to control the expression of SlaR.

### Optimization of culture conditions and medium

**Fig. 4:**
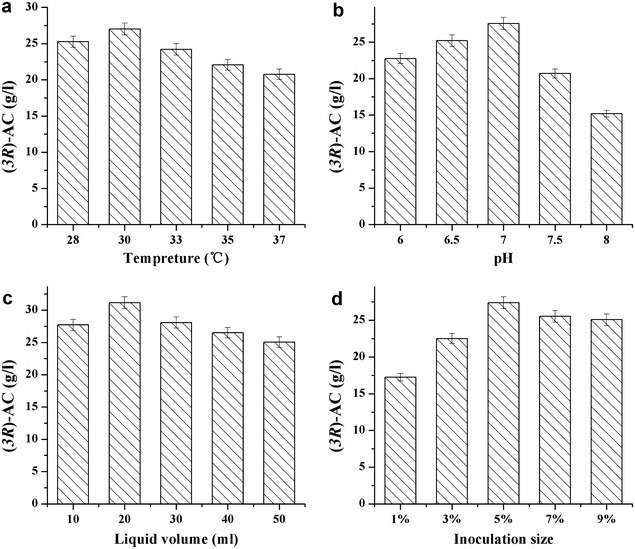
Effects of different culture conditions on (*3R*)-AC production by *Serratia marcescens* MG15. **a** Temperature; **b** pH; **c** liquid volume; **d** inoculation size

To further improve the (*3R*)-AC production of strain MG15, the fermentation conditions and medium composition were optimized. As shown in Fig. 4, the (*3R*)-AC production was highest at 30 °C, pH 7.0, 20 ml liquid volume, and 5% inoculation size. Then the above conditions were used for optimizing the medium composition.

Plackett–Burman design was first used to screen significant factors from the seven variables including sucrose, yeast extract, sodium citrate, sodium acetate, NH_4_H_2_PO_4_, MgSO_4_, and MnSO_4_, based on preliminary experiments and related reports. The design details and the corresponding results are presented in Table 3. Analysis to the experimental data showed that there was a wide variation of (*3R*)-AC production from 19.78 to 32.31 g/l in the 12 experiments. After analysis of the regression coefficients, sucrose, yeast extract, sodium citrate, and NH_4_H_2_PO_4_ showed positive effects, while sodium acetate, MgSO_4_, and MnSO_4_ showed negative effects. The *p* values of sucrose and sodium acetate were >0.05, so they were considered significant, whereas the other factors were considered insignificant. Although sucrose and sodium acetate were identified to be two significant medium components, the optimal levels of them were still need to be determined in further experiments.

Sucrose and sodium acetate were further optimized based on the results of PB design using CCD. The experimental design and results are shown in Table 4. After analyzing the experimental data, the following second-order polynomial equation was obtained, describing the relationship between the (*3R*)-AC production (*Y*) and the concentrations of sucrose (*X*_1_) and sodium acetate (*X*_2_).

$$\eqalign{
  & Y =  - 4.17 + 0.702{X_1} + 2.06{X_2} - 0.002744X_1^2  \cr 
  & \qquad \; - 0.770X_2^2 - 0.0089{X_1}{X_2} \cr} $$

The *R* ^2^ value of this model was 0.9793, indicating the model could explain 97.93% of the variability in the response. The adjusted *R* ^2^ was 96.45%, which indicated a high significance of the model.

**Fig. 5:**
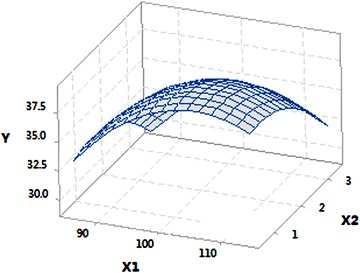
Response surface figure of the mutual effects of sucrose (*X*_*1*_) and sodium acetate (*X*_*2*_) on (*3R*)-AC production

The three-dimensional response surface (Fig. 5) was also used to determine the effects of sucrose and sodium acetate on (*3R*)-AC production. It was obvious that the response surface was convex in nature, suggesting that the optimum conditions were well defined. Based on the equation model and the response surface, the optimal concentration of sucrose and sodium acetate was 105 and 1 g/l, respectively; the corresponding maximum (*3R*)-AC production was predicted to be 39.27 g/l.

**Fig. 6:**
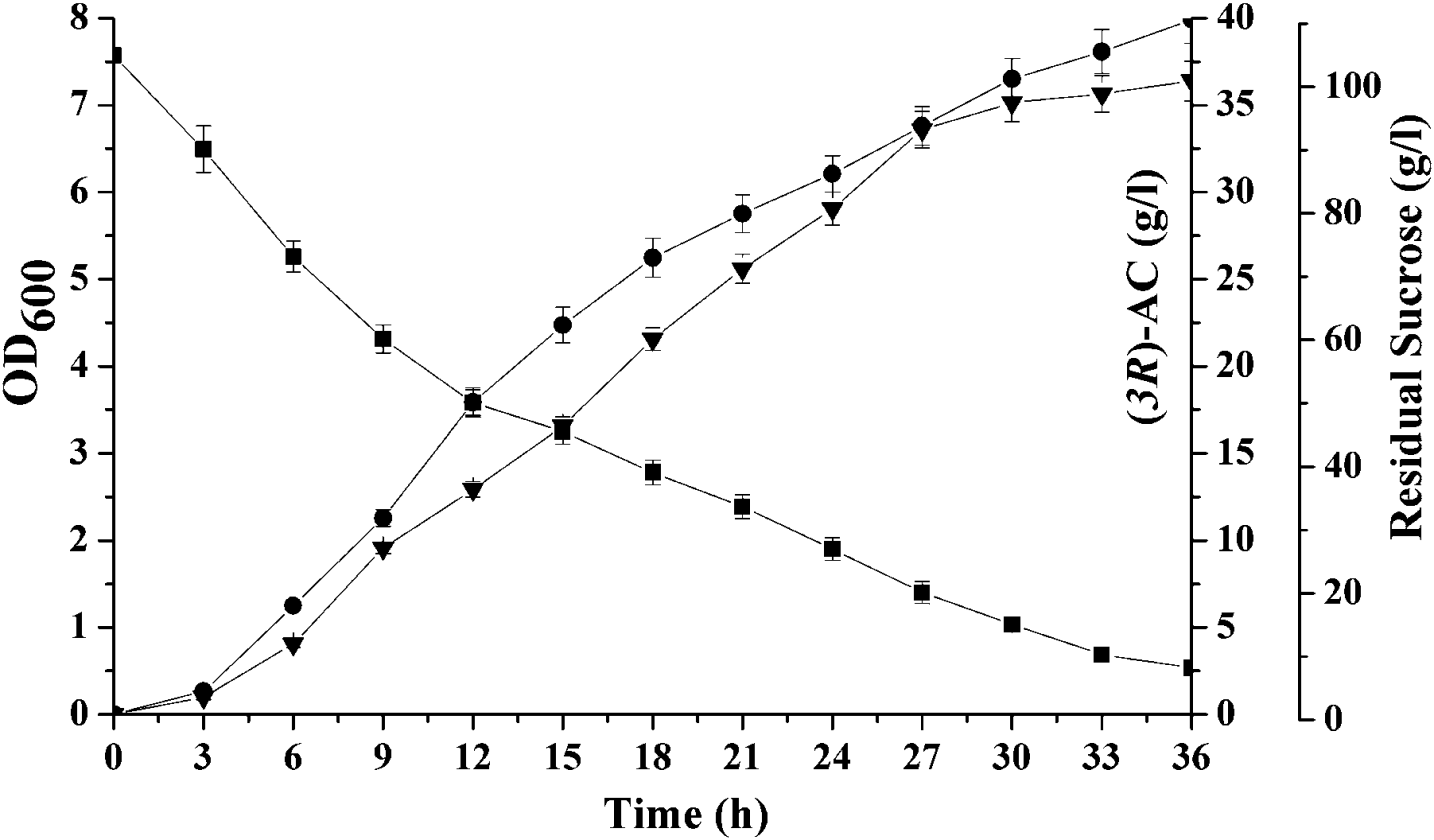
Time course of (*3R*)-AC production of strain MG15 in the suitable culture conditions and the optimized medium. OD_600_ (*filled up-right triangles*), residual sucrose (*filled squares*) and (*3R*)-AC (*filled circles*)

From the above results, the optimum medium composition for (*3R*)-AC production by *S. marcescens* MG15 was as follows (g/l): sucrose 105, yeast extract 25, sodium citrate 15, sodium acetate 1, NH_4_H_2_PO_4_ 0.5, MgSO_4_ 0.2, and MnSO_4_ 0.02. Validation experiment was carried out in triplicate test to confirm the reliability of the model equation. As shown in Fig. 6 and Table 6, the average (*3R*)-AC yield of 39.91 ± 1.35 g/l was obtained under the optimized condition, which was very close to the predicted maximum value of 39.27 g/l. Besides, the conversion rate was 80.13% and the productivity was 1.11 g/l h. Therefore, this result indicated the validity of the model.

## Conclusion

In this study, the *sla*C gene and *gld*A gene responsible for reducing AC to 2,3-BD were knocked out, and the resulting strain MG14 was found to produce a large amount of optically pure (*3R*)-AC, with a little 2,3-BD. In addition, overexpression of the transcription regulator SlaR under the control of the P_C_ promoter was successful for enhancing (*3R*)-AC synthesis by *S. marcescens* MG14. Furthermore, the suitable culture conditions were optimized using single-factor experiments and the optimal medium component was developed using PB design and RSM. Based on the suitable culture conditions and the optimized medium, the engineered *S. marcescens* MG16 could produce 39.91 ± 1.35 g/l (*3R*)-AC with a conversion rate of 80.13% and productivity of 1.11 g/l h in the flask fermentation.

## Abbreviations

AC: acetoin
*meso*-2,3-BDH: meso-2,3-butanediol dehydrogenase
GDH: glycerol dehydrogenase
2,3-BD: 2,3-butanediol
α-ALS: α-acetolactate synthase
α-ALDC: α-acetolactate decarboxylase
DA: diacetyl
AR: AC reductase
FAD: flavin adenine dinucleotide
TPP: thiamine pyrophosphate
LB: Luria–Bertani
PB: Plackett–Burman
RSM: response surface methodology
CCD: central composite design
